# Supplementary figures and images for: Prior physical illness predicts death better than acute physiological derangement on intensive care unit admission in COVID-19: A Swedish registry study
Source: PLoS One. 2023 Sep 27;18(9):e0292186. doi: 10.1371/journal.pone.0292186 (PMC10529545; doi:10.1371/journal.pone.0292186)

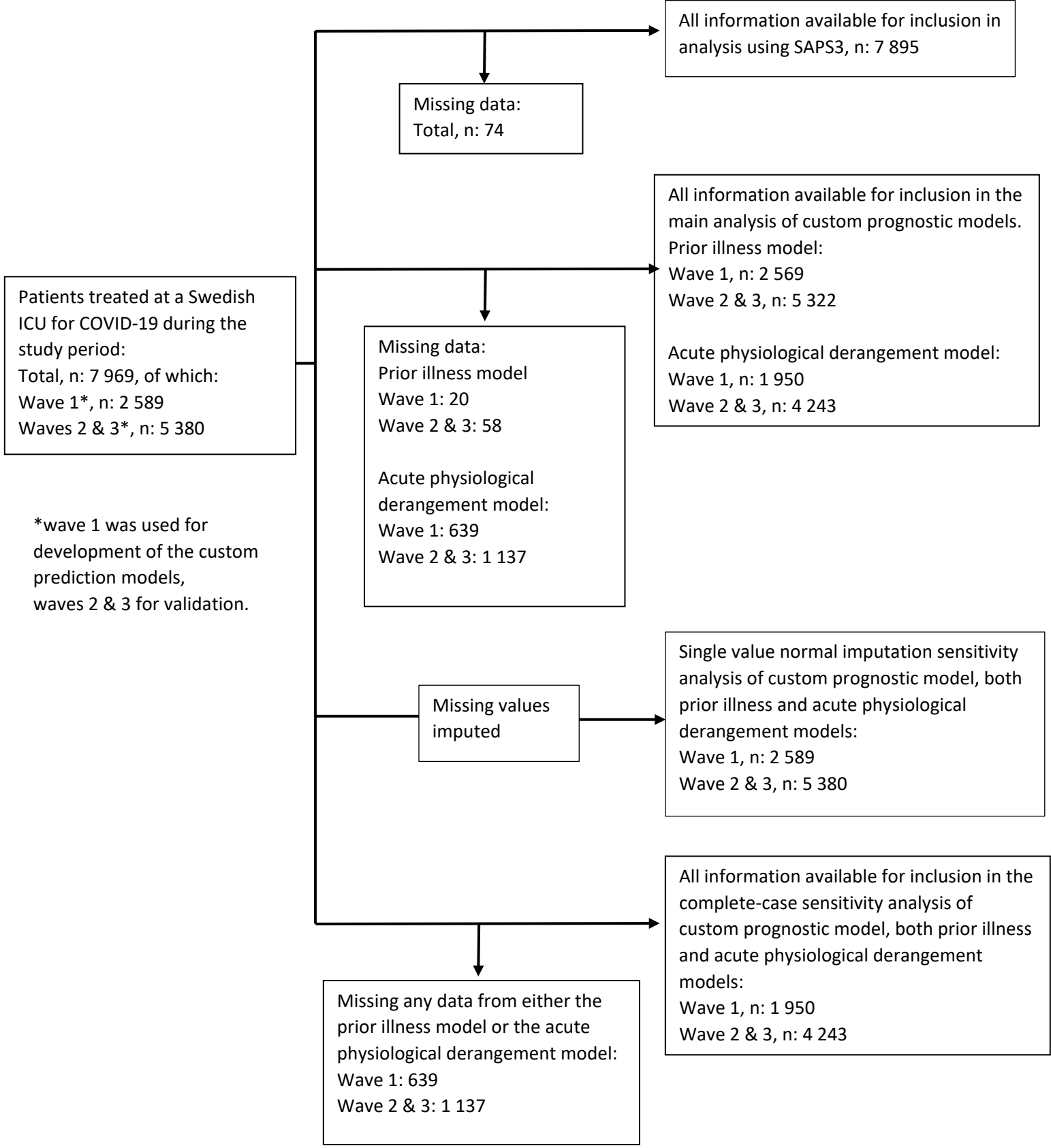

Supplement: S1 Fig — (PDF) [file pone.0292186.s001.pdf]

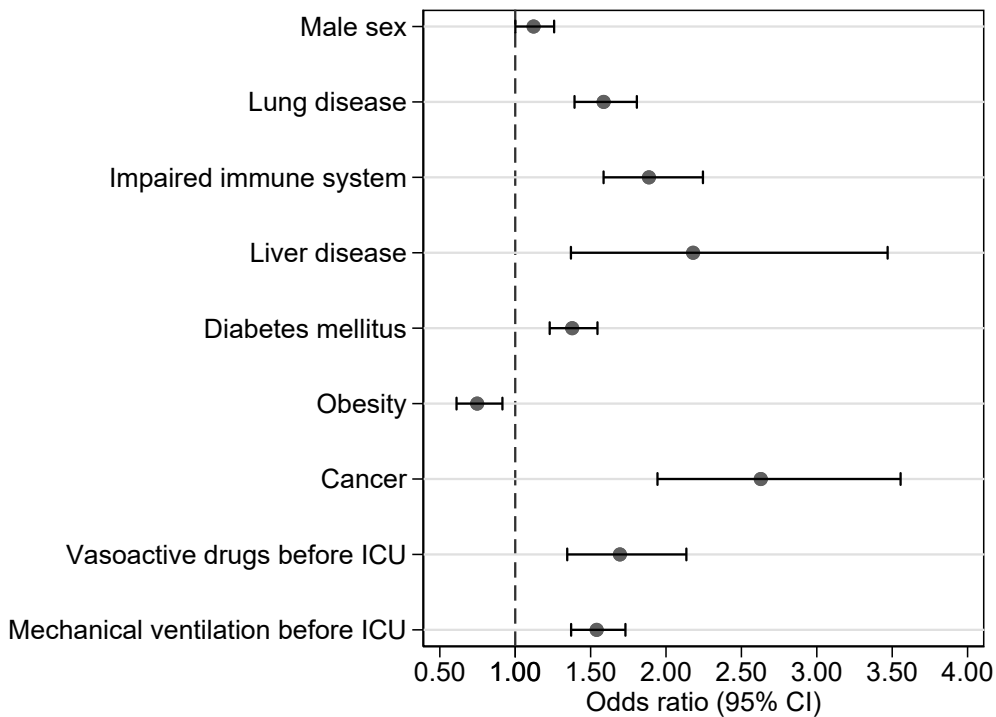

Supplement: S2 Fig — (PDF) [file pone.0292186.s002.pdf]

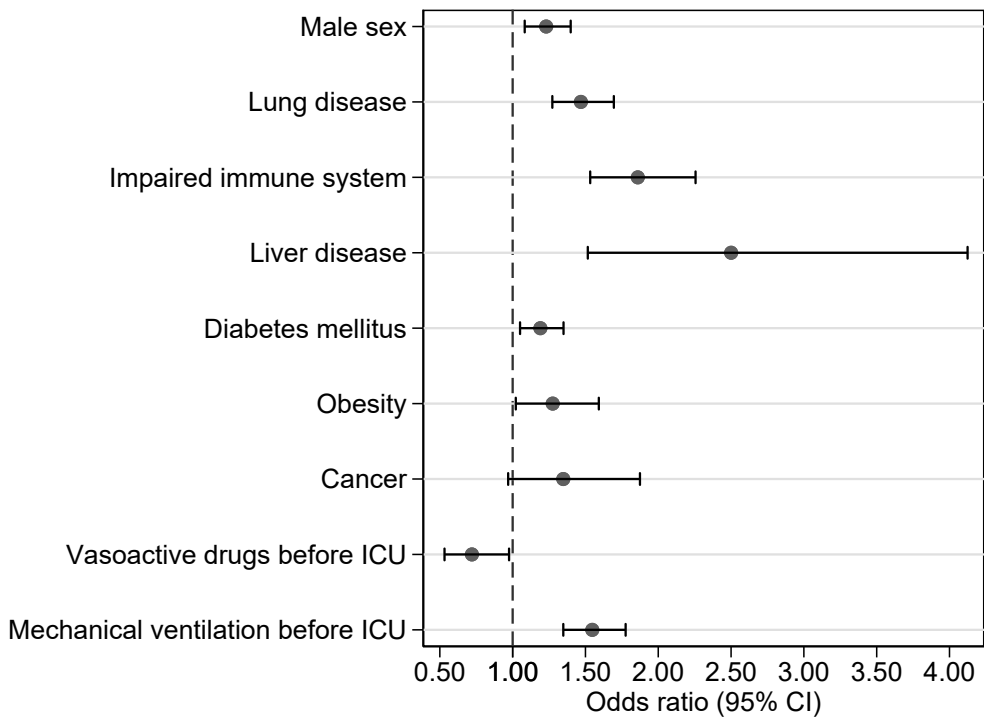

Supplement: S3 Fig — Predictors adjusted for all other variables in the respective model: sex, chronic lung disease, impaired immune system, chronic liver disease, diabetes, obesity and cancer adjusted for each other and age. Vasoactive medication prior to ICU admission and mechanical ventilation before ICU adjusted for each other and highest body temperature, lowest systolic blood pressure, highest bilirubin, highest creatinine, highest leucocyte count, lowest thrombocyte count, lowest pH, lowest PaO2, number of symptomatic days prior to ICU admission and days in hospital prior to ICU admission. (PDF) [file pone.0292186.s003.pdf]

Odds ratio (95% CI) for 30-day mortality

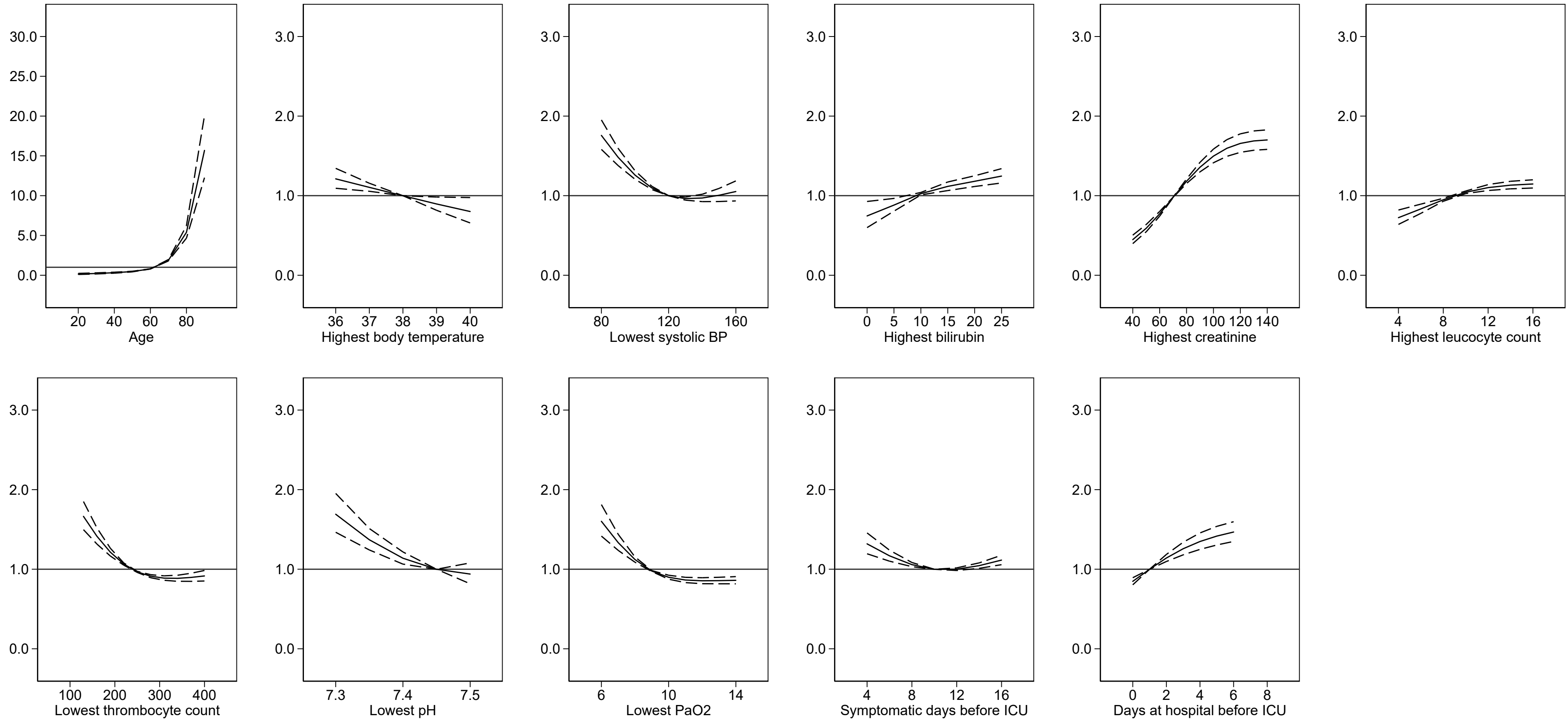

Supplement: S4 Fig — Presented using restricted cubic splines with knots at the 10th, 50th and 90th percentile. (PDF) [file pone.0292186.s004.pdf]

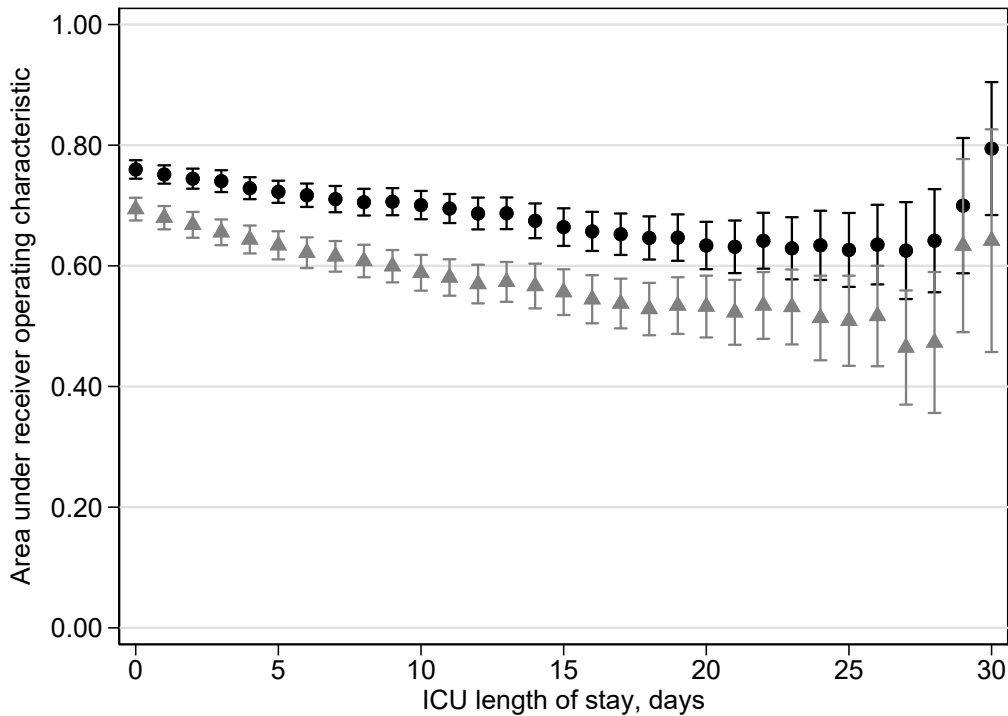

Supplement: S5 Fig — Prior illness: black circles, acute physiological derangement: grey triangles. AUC: area under the receiver operating characteristic. (PDF) [file pone.0292186.s005.pdf]

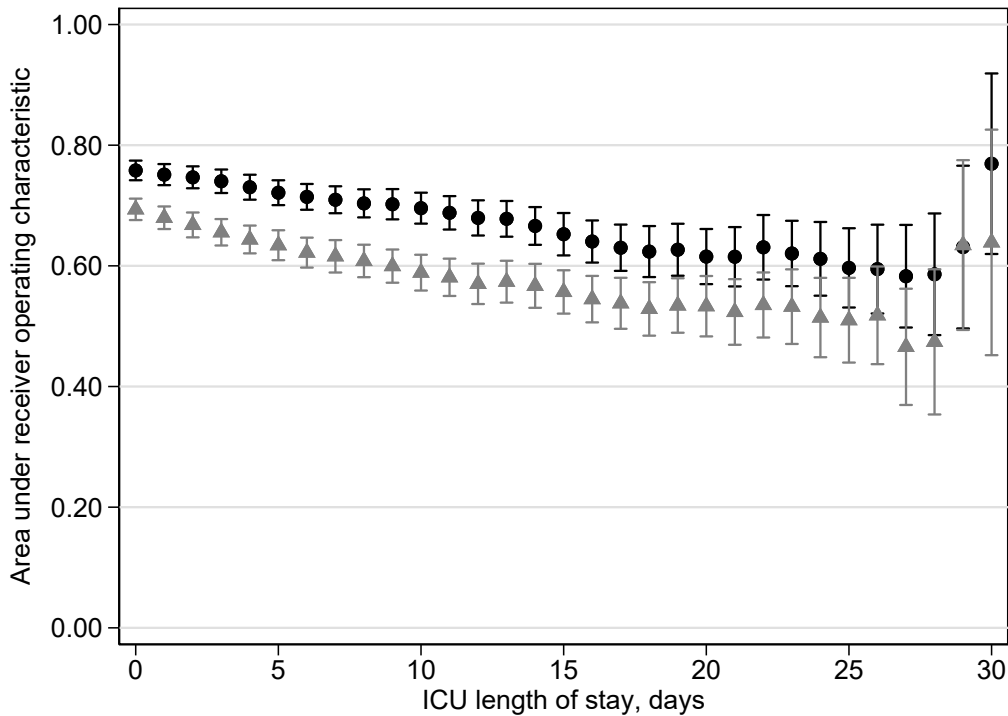

Supplement: S6 Fig — Only includes complete cases with all information necessary to be included in both the prior and acute models. Prior illness: black circles, acute physiological derangement: grey triangles. AUC: area under the receiver operating characteristic. (PDF) [file pone.0292186.s006.pdf]

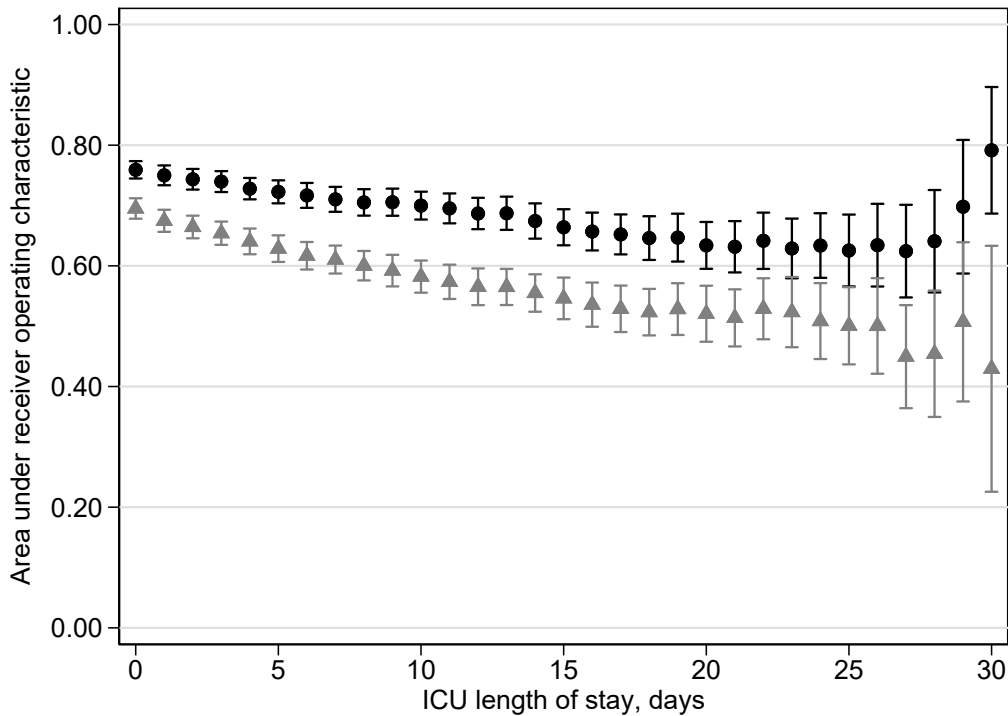

Supplement: S7 Fig — Missing data replaced with single imputation of a normal value. Prior illness: black circles, acute physiological derangement: grey triangles. AUC: area under the receiver operating characteristic. (PDF) [file pone.0292186.s007.pdf]
