# Supplementary material for: Prior physical illness predicts death better than acute physiological derangement on intensive care unit admission in COVID-19: A Swedish registry study
Source: PLoS One. 2023 Sep 27;18(9):e0292186. doi: 10.1371/journal.pone.0292186 (PMC10529545; doi:10.1371/journal.pone.0292186)
Supplement: Attachment — Submitted filename: Response to reviewers.docx [file pone.0292186.s011.docx]

*Dear Editor and Reviewers,*

*Thank you for your time and expertise helping us improve our article. We have addressed all comments, we hope satisfactorily. Please see attached manuscript with changes highlighted.*

*Sincerely,*

*The authors, through Karl Stattin*

Please review your reference list to ensure that it is complete and correct. If you have cited papers that have been retracted, please include the rationale for doing so in the manuscript text, or remove these references and replace them with relevant current references. Any changes to the reference list should be mentioned in the rebuttal letter that accompanies your revised manuscript. If you need to cite a retracted article, indicate the article’s retracted status in the References list and also include a citation and full reference for the retraction notice.

Additional Editor Comments:
Thanks you for the revisions which the reviewer has seen and commented on. The review is almost complete. Kindly attend to the reviewer's requirements CAREFULLY, ensuring that no new problems arises. Please note that Plos1 limits the number of reviews to 2 and that this is the final review round.
All the best

*Thank you for the opportunity to review our manuscript. References have been updated in order to adhere to the PLOS ONE style guidelines. None of the cited references have been redacted.*

Reviewer #1: Future submission:
Please ensure that your manuscript meets PLOS ONE's style requirements.
Please specify where the minimal data set underlying the results described in your manuscript can be found.

*Thank you, we will be more careful to follow style requirements. Availability of the minimal dataset has been added to the paragraph “Ethical considerations” line 87-91: The entire dataset is available to researchers upon request with appropriate ethical approval from the Swedish Intensive Care Registry, at [www.icuregswe.org](http://www.icuregswe.org). Swedish law does not support sharing of individual level registry data, and thus our ethical approval from the Swedish Ethical Review Authority does not support public uploading of individual-level data as it contains sensitive patient information.*

Reviewer #2: Lines 136-137: The authors mentioned in response to comment number 7 of Reviewer 2, that mechanical ventilation only refers to invasive mechanical ventilation and that the manuscript has been edited to specify this. However, I am unable to note this in the revised manuscript.

The revised manuscript shows significant improvements and no further major revisions are proposed. This original research addresses a relevant and important question that is clearly answered by the results and discussion, with conclusions supported by the data.

*Thank you for your thorough revision. We apologise for the omission. Line 140 has been edited to specify invasive mechanical ventilation.*
