# Supplementary material for: Prior physical illness predicts death better than acute physiological derangement on intensive care unit admission in COVID-19: A Swedish registry study
Source: PLoS One. 2023 Sep 27;18(9):e0292186. doi: 10.1371/journal.pone.0292186 (PMC10529545; doi:10.1371/journal.pone.0292186)
Supplement: Attachment — Submitted filename: Reviewer comments.docx [file pone.0292186.s008.docx]

**Reviewer comments**

1. In the Background section of the abstract, the authors mention that this “…has not been studied in COVID-19” (line 27) but mentions in line 214 in the Discussion that “Earlier studies evaluating SAPS3 in COVID-19 have shown acceptable AUC values ranging from 0.69-0.83, which is consistent with our findings.” Are the authors suggesting that these referenced studies are different? Clarification is required in terms of how this study is different from others or has not been studied in COVID-19.
2. In the Methods section of the abstract (line 33) the authors use AUC ROC as the abbreviation for “area under receiver operating characteristic” but later in the text only AUC is used as an abbreviation. Suggest to use only one abbreviation if referring to one concept, alternatively new abbreviations need to be clarified/explained.
3. Please clarify the result section of abstract (lines 39 - 41): do these two AUC values refer to the Box1 or Box3 components of SAPS3 and/or the trained results on wave-1 patients and tested results on wave-2/3 patients. What are the two variables compared with each other?
4. Lines 85 – 89: Please provide a heading for the ethics statement under the Method section e.g. “Ethical considerations”
5. The method section requires refinement and sufficient detail for better understanding.
   a. The total sample size (entire cohort) – patients admitted to ICU between the given dates (?n=7969) – The authors only mention this in the Results section and not in the Method section
      i. The authors are unclear (lines 138-146) on how n=2569 for the prior illness model in the development dataset and n=5322 in the validation dataset was selected. Please clarify the exclusion and inclusion criteria for each dataset as well as for each model. (The authors should provide the eligibility criteria, and the sources and methods of selection of participants in the method section)
      ii. Following the sensitivity analyses and exclusion of individuals, the development data set was n=1950 and the validation data set was n=4243 for both models
      iii. A flow diagram/chart may help with clarification or better presentation of each stage of the number of individuals/participants and how the authors got to this final cohort that is being used for the statistical analysis
      iv. The authors do not mention that wave-1 patients are being used for the development data set and wave-2/3 patients being used for the validation data set in the Method section.
          1. The following is mentioned in line 40 of the abstract only: “…custom logistic models trained on wave-1 patients (n=?) and tested in wave-2 and -3 (n=?) patients.” Does this refer to development and validation data sets respectively?
      v. Report numbers of individuals at each stage of the study and reasons for non-participation/exclusion at each stage of the study
   b. The authors mention that two prognostic models were compared: 1. Prior physical illness (? chronic model/?Box1) and 2. Acute physiological derangement (?acute model/Box3)
      i. Later, in the text these models are alternatively referred to as acute and chronic models (lines 198 and 199). The authors are advised to clarify this in the method section and/or only use one term to describe a model to avoid confusion later in the text.

SUGGEST: revise the Method section

6. Lines 154-155 “Patients scored a median of 17 points (IQR 13 to 21) on Box 1 (of which median 9 (IQR 5 to 13**) from age**) and 10 (IQR 5 to 14) points on Box 3 of SAPS3.”: This sentence seems to have some missing information and values are not available in Table 1 to find out. Please also explain what these values mean and what is their significance.
   a. Please use alternative brackets “[]” within another set of brackets.
7. Line 157: Does “mechanical ventilation” refer to all patients that were mechanically ventilated but not necessarily intubated?
8. Line 157-158: “During ICU stay 1 566 (19.7%) patients died whereas 1 878 (23.6%) died within 30 days of ICU admission”
   a. I am not sure what the authors are trying to say with these numbers.
   b. Please explain how the total number of patients who died during the ICU stay (n=1566) is less than the number of patients who died within 30 days of ICU admission (n=1878). Does “ICU stay” refer to a longer period than the 30 days that are mentioned in line 105?
   c. Line 105: “Patients were followed for 30 days after ICU admission for mortality.” And because “…hospital mortality was not available, 30-day mortality was chosen as the outcome.” (line 111)
   d. Are these values (percentages) proportions of the total cohort, even if they were not included in the statistical analysis?
9. Line 166: Please provide AUC values and their precision (95% confidence intervals) for the statement that there was no difference in discrimination between waves.
10. Line 168-169: The calibration belt graph shown in Figure 2 does not clearly illustrate the finding
    a. Please clarify this with clear Y-axis and X-axis titles and values in the text.
11. Line 171: Please indicate that this is referring to Figure 1, if this is so.
12. Line 180: SAPS3 Box 1 predicted death better than Box 3 **at all times** during ICU stay
    a. Are there statistically significant differences between Box1 and Box3 after day 20 when the 95% confidence intervals overlap to make this statement “at all times” ?
    b. Please provide statistical justification for this statement.
13. Are the values shown in Figure 1 and Figure 3 results from the development dataset or the validation dataset? Please clarify if this is indeed based on the development dataset.
14. Lines 186-192: Consider this information in the Method section rather than under “Results”.
    a. Describe how the two models were developed and the selection of participants in the method section.
15. Line 195: Is there a statistically significant difference between the two models beyond day 20? Please justify or clarify this statement.
    a. Please provide numerical results in addition to the graphs for the AUC of ICU length of stay at 30 days.
    b. This is similar to the point made in no 12.
16. Line 219: Please consider the following article and reconsider the statement: “No previous study has compared…”

Ana Paula Pires Lázaro, Polianna Lemos Moura Moreira Albuquerque, Gdayllon Cavalcante Meneses, et al. Critically ill COVID-19 patients in northeast Brazil: mortality predictors during the first and second waves including SAPS 3, *Transactions of The Royal Society of Tropical Medicine and Hygiene*, Volume 116, Issue 11, November 2022, Pages 1054–1062, <https://doi.org/10.1093/trstmh/trac046>

17. Line 224: Repetition of lines 205-206 or is this a different statement based on a different data set? Please clarify.

This is an interesting article with good results that should be considered for publication after the major and minor changes suggested.
